# Early Prostate-Specific Antigen (PSA) Change at Four Weeks of the First-Line Treatment Using Abiraterone and Enzalutamide Could Predict Early/Primary Resistance in Metastatic Castration-Resistant Prostate Cancer

**DOI:** 10.3390/cancers13030526

**Published:** 2021-01-30

**Authors:** Taizo Uchimoto, Kazumasa Komura, Wataru Fukuokaya, Takahiro Kimura, Kazuhiro Takahashi, Kazuki Nishimura, Keita Nakamori, Yuya Fujiwara, Tomohisa Matsunaga, Takeshi Tsutsumi, Takuya Tsujino, Ryoichi Maenosono, Yuki Yoshikawa, Kohei Taniguchi, Tomohito Tanaka, Hirofumi Uehara, Naokazu Ibuki, Hajime Hirano, Hayahito Nomi, Kiyoshi Takahara, Teruo Inamoto, Shin Egawa, Haruhito Azuma

**Affiliations:** 1 Department of Urology, Osaka Medical College, Osaka 569-8686, Japan; uro053@osaka-med.ac.jp (T.U.); uro081@osaka-med.ac.jp (K.N.); uro076@osaka-med.ac.jp (K.N.); uro072@osaka-med.ac.jp (Y.F.); uro065@osaka-med.ac.jp (T.M.); uro070@osaka-med.ac.jp (T.T.); uro061@osaka-med.ac.jp (T.T.); uro064@osaka-med.ac.jp (R.M.); uro066@osaka-med.ac.jp (Y.Y.); uro040@osaka-med.ac.jp (H.U.); uro041@osaka-med.ac.jp (N.I.); uro052@osaka-med.ac.jp (H.H.); uro022@osaka-med.ac.jp (H.N.); tinamoto@osaka-med.ac.jp (T.I.); uro001@osaka-med.ac.jp (H.A.); 2 Translational Research Program, Osaka Medical College, Osaka 569-8686, Japan; sur144@osaka-med.ac.jp (K.T.); gyn123@osaka-med.ac.jp (T.T.); 3 Department of Urology, The Jikei University School of Medicine, Tokyo 105-8461, Japan; fukuokaya@jikei.ac.jp (W.F.); h19ms-takahashi@jikei.ac.jp (K.T.); s-egpro@jikei.ac.jp (S.E.); 4 Department of Urology, Fujita-Health University School of Medicine, Nagoya 470-1192, Japan; takahara@fujita-hu.ac.jp

**Keywords:** castration-resistant prostate cancer, androgen signaling inhibitors, abiraterone acetate, enzalutamide, prostate-specific antigen, PSA decline, early PSA

## Abstract

**Simple Summary:**

Serum prostate-specific antigen (PSA) level is the most valuable biomarker in prostate cancer. This study investigates the predictive value of achieving >30% PSA decline at four weeks of first-line androgen signaling inhibitors (ASIs) using a multi-institutional cohort dataset of 254 mCRPC patients. The achievement of >30% PSA decline at four weeks is an independent predictor for overall survival (OS). Interestingly, in patients who did not achieve >30% PSA decline at four weeks—an achievement of the >30% PSA decline at 12 weeks is eventually observed in 30.9% of those patients. To identify the variables that discriminate the patient survival in 97 patients without achieving >30% PSA decline at four weeks of the first-line treatment, a multivariate analysis is conducted. The duration of androgen deprivation therapy before CRPC < 12 months and Eastern Cooperative Oncology Group Performance Status ≥ 1 are identified as independent predictors for shorter OS for those patients.

**Abstract:**

The identification of early or primary resistance to androgen signaling inhibitors (ASIs) is of great value for the treatment of metastatic castration-resistant prostate cancer (mCRPC). This study evaluates the predictive value of prostate-specific antigen (PSA) response at dour weeks of first-line ASIs treatment for mCRPC patients. A total of 254 patients treated with ASIs (abiraterone acetate: AA and enzalutamide: Enz) at the first-line treatment are retrospectively analyzed. Patients are stratified according to the achievement of >30% PSA decline at 4 and 12 weeks from the treatment initiation. At four weeks of the treatment, 157 patients (61.8%) achieved >30% PSA decline from the baseline. Thereafter, 177 patients (69.7%) achieved >30% PSA decline at 12 weeks of the treatment. A multivariate analysis exhibits >30% PSA decline at four weeks as an independent predictor for overall survival (OS). We note that 30 of 97 (30.9%) patients who did not achieve >30% PSA decline at four weeks consequently achieved >30% PSA decline at 12 weeks, and had a comparable favorable three years OS rate as the 147 patients achieving >30% PSA decline at both 4 and 12 weeks. To identify the variables that discriminate the patient survival in 97 patients without achieving >30% PSA decline at four weeks, a multivariate analysis is performed. The duration of androgen deprivation therapy before CRPC ≤ 12 months and Eastern Cooperative Oncology Group Performance Status ≥ 1 are identified as independent predictors for shorter OS for those patients. These data offer a concept of early treatment switch after four weeks of first-line ASIs when not observing >30% PSA decline at four weeks—particularly in patients with a modest effect of ADT and poor performance status.

## 1. Introduction

Prostate cancer (PC) is the most common cancer in men, and estimated numbers in the United States account for 31,620 deaths and 174,650 new patients in 2019 [1]. Most PC patients are diagnosed at the local stage, and five years overall survival (OS) rate in those patients reached ≥95% with the established treatment strategy, while patients with distant metastasis declined to that of 30% [2]. Androgen deprivation therapy (ADT) has been a central mainstay of the treatment for advanced PC. Although almost all PC patients initially respond to ADT, acquired resistance has been developed in most patients within a couple of years, namely, castration-resistant PC (CRPC), which leads to the lethal stage through multiple mechanisms [3,4,5,6,7]. In the last decade, several new drugs approved by FDA have emerged with improved clinical outcomes in metastatic CRPC (mCRPC) patients, including androgen signaling inhibitors (ASIs: abiraterone, enzalutamide, and apalutamide), cabazitaxel, and radium-223 [8,9,10,11,12,13]. Those new agents have innovated on the treatment strategy for mCRPC, which now raises new questions, i.e., the precise treatment sequence and uncovering a reliable marker to predict the treatment outcomes using those drugs for mCRPC patients [14,15,16].

Prostate-specific antigen (PSA) is widely used for diagnosis and evaluation of treatment effect for PC. It has been well-documented that PSA kinetics from the treatment initiation would serve as a predictive surrogate for OS [17,18]. Most prospective trials have adopted an evaluation of PSA at 12 weeks rather than four weeks of the treatment, due to the possibility of late response and flare reactions, especially when using taxanes [19,20,21]. Thus, a hypothesis that an early PSA response as early as four weeks of the treatment offers a predictive impact on treatment outcomes is still controversial. Recently, a report from an international study exhibited that >30% PSA decline (PSA30D) at four weeks of the treatment using abiraterone acetate (AA) and enzalutamide (Enz) is strongly associated with OS, whereas their cohort included the population of pre- and post-chemotherapy patients [22]. In the present study, we assessed the predictive value of PSA30D at four weeks focusing on the first-line ASIs treatment.

## 2. Materials and Methods

### 2.1. Cohorts and Variables

**Figure 1:**
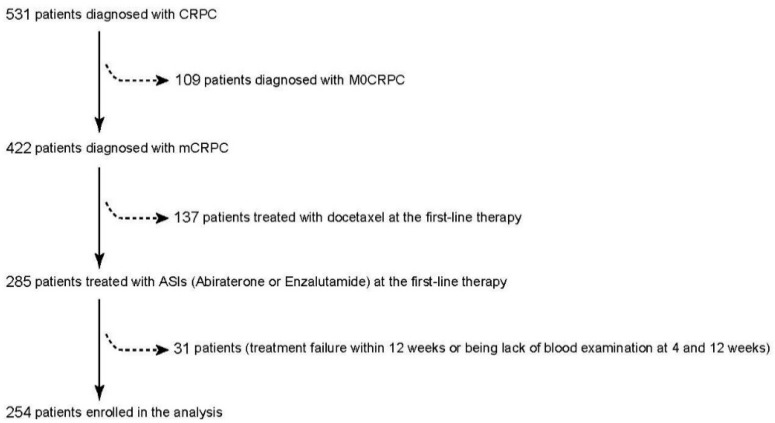
Study design in the current study. A total of 254 mCRPC patients treated with first-line abiraterone acetate (AA) or enzalutamide (Enz) were analyzed.

This retrospective study was conducted using multi-institutional cohorts, including Osaka Medical College (Osaka, Japan) and The Jikei University School of Medicine (Tokyo, Japan) with their local satellite hospitals. The design of the study has been approved by the institutional review board of Osaka Medical College (IRB approval number: RIN-750-2571, Date of approval: 24 January 2020) and performed with the consent of the World Medical Association Declaration of Helsinki [23]. As shown in Figure 1, clinical records of consecutive 531 patients were collected, in which 422 (79.5%) patients were diagnosed as mCRPC with any distant metastasis, and 109 (20.5%) patients were assigned to non-metastatic CRPC (nmCRPC) with no distant metastasis at the diagnosis of CRPC. In the present study, we excluded nmCRPC patients from the analysis. Of 422 mCRPC patients, patients were treated with first-line therapy, including abiraterone acetate (AA) (*n* = 134, 31.8%), enzalutamide (Enz) (*n* = 151, 35.8%), and docetaxel (137, 32.4%). Of the patients treated with ASIs at the first-line treatment (AA and Enz), 31 patients experienced treatment failure within 12 weeks or did not have records of serum PSA level at both 4 and 12 weeks. In total, 254 mCRPC patients treated with ASIs (AA and Enz) at the first-line treatment were enrolled in the present study to evaluate clinical outcomes and PSA kinetics.

Clinical variables in the present study involve Age at CRPC (years) (≤70/>70), PSA (ng/ml) at diagnosis, Clinical stage at diagnosis (T2-T3/T4), Gleason sum score at diagnosis (<9/≥9), duration of ADT before CRPC (months) (≥12/<12), bone mets at CRPC (−/+), lymph node mets at CRPC (−/+), visceral mets at CRPC (−/+), eastern cooperative oncology group performance status (ECOG-PS) at CRPC (0/≥1). The data of blood examination includes PSA at the diagnosis of CRPC, alkaline phosphatase (ALP), lactate dehydrogenase (LDH), and hemoglobin (Hb). The PSA value was measured by automated Chemiluminescence Immunoassays (CLIA) (ARCHITECT Total PSA Calibrators 7K70-01, ABBOTT Laboratories) in all participating institutes. Other laboratory data were measured by the methods of enzyme-linked immunosorbent assay (ELISA) (FUJIFILM Toyama Chemical Co. Ltd, Miyazaki 889-1601, Japan; LDH: catalog number 468-76691, ALP: catalog number 464-55791).

### 2.2. Diagnosis of CRPC, Follow-Up, and Treatments

CRPC was defined according to the PCWG2 guidelines, i.e., serum testosterone level <50 ng/dL with either PSA progression (an increase of 25% and an absolute increase of 2 ng/mL or more above PSA nadir) or radiographic progression [24]. The clinical-stage was evaluated using magnetic resonance imaging (MRI), computed tomography (CT), and bone scintigraphy to examine the number and location of metastasis at the diagnosis of CRPC. Lymph node metastasis was defined with >15 mm according to the RECIST guidelines (version 1.1) [25]. All the other clinical variables, including age, response duration of ADT, Gleason sum score at diagnosis, ECOG-PS, and clinical laboratory measurement in peripheral blood (PSA, ALP, LDH, and Hb), were recorded at the diagnosis of CRPC. ASIs were administrated with a standard dose, i.e., Enz in 160 mg/day [8,10] and AA in 1000 mg plus prednisone 10 mg/day. Dose modification and treatment intervals were considered by side-effects and general conditions with the physician’s discretion. Follow-up CT for detecting any findings suspected of disease progression was scheduled every three months from the diagnosis of CRPC. MRI, bone scintigraphy, and positron emission tomography/computed tomography (PET/CT) were further performed when necessary for the definitive diagnosis of disease progression. The primary endpoints of the study involved OS. The secondary endpoints were time to PSA progression (TTPP), which was defined based on the PCWG2 guidelines (an increase of 25% and an absolute increase of 2 ng/mL or more above the PSA nadir) [24]. OS was calculated from the diagnosis of mCRPC to the last follow-up or death. Serum PSA level was measured every month from the baseline assessment at the initiation of the first-line treatment.

### 2.3. Statistical Analyses

A Chi-square test was performed to evaluate the distribution of each variable by a contingency table. The student’s t-test was examined to assess the difference between the variables. Kolmogorov-Smirnov normality was conducted to check normal distribution in continuous variables. For variables with non-normal distribution, Wilcoxon or Kruskal-Wallis test was evaluated to examine the difference between the groups. Kaplan-Meier curves were calculated to estimate the survival ratio. The ability for outcome prediction of continuous variables in PSA changes was determined by receiver operating characteristic (ROC) curve analysis, and the optimal cut-off values were defined by the Youden index as the point maximizing the difference between true positive rate and false-positive rate across all possible cut-point values [26,27]. A log-rank test was performed to define the clinical difference between categorized groups. On multivariate analysis to examine the association of variables with OS, Cox proportional-hazard regression models were utilized to define covariate-adjusted hazard ratios (HR). All the statistical tests were two-sided with *p* < 0.05 considered to delineate statistical significance, which was performed using JMP^®^ 13 (SAS Institute Inc., Cary, NC, USA) and GraphPad Prism software (GraphPad Software, La Jolla, CA, USA).

## 3. Results

The background of the cohort and clinical characteristics in the present study are shown in Figure 1 and Table 1. The mean age at diagnosis of CRPC was 73 years old. There were 66 of 254 patients (26.0%) who deceased during the follow-up. The median follow-up duration for patients who were alive (188 patients) and had died (66 patients) were 18 and 13 months, respectively. All the baseline backgrounds at the diagnosis of CRPC were comparable between the first-line treatment groups (AA and Enz) (Table 1), and there was no significant difference between AA and Enz in OS, TTPP from the initiation of the first-line treatment.

**Figure 2:**
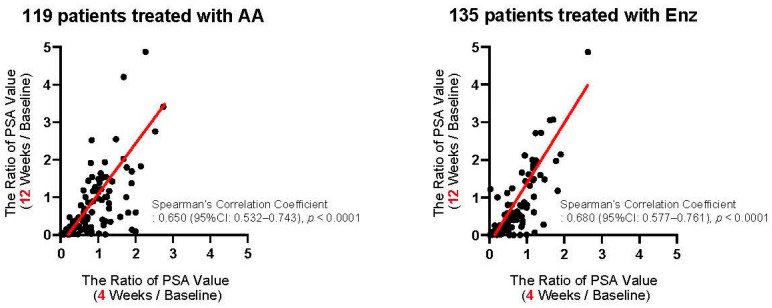
Correlation of PSA changes between 4 and 12 weeks from the initiation of the first-line abiraterone acetate (AA: left panel) and enzalutamide (Enz: right panel). The axis indicates the ratio of PSA value, i.e., (at four weeks/at baseline: X-axis) and (at 12 weeks/at baseline: Y-axis).

**Figure 3:**
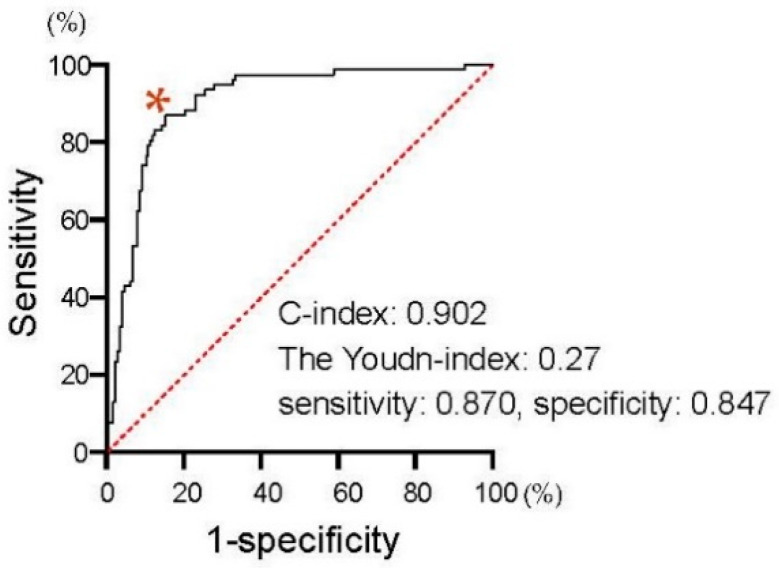
Receiver operating characteristic (ROC) curves to determine the optimal cut-off in PSA changes at four weeks of the treatment for the event of achieving PSA30D at 12 weeks. The Youden-index, the point maximizing the difference between the true-positive and false-positive rates across all possible cut-off values, was indicated at * mark.

We first assessed the correlation of PSA changes between 4 and 12 weeks from the baseline. As shown in Figure 2, PSA changes at four weeks exhibited a significant positive correlation with those at 12 weeks from the initiation of the first-line treatment in patients treated with both AA (Spearman’s correlation coefficient: 0.650, 95%CI: 0.532–0.743, *p* < 0.0001) and Enz (Spearman’s correlation coefficient: 0.680, 95%CI: 0.577–0.761, *p* < 0.0001). We investigated the ROC curve to determine the optimal cut-off in PSA changes at four weeks of the treatment for the event of achieving >30% PSA decline (PSA30D) at 12 weeks. As shown in Figure 3, the Youden-index, the point maximizing the difference between the true-positive and false-positive rates across all possible cut-off values, identified the optimal cut-off value of PSA change from the baseline at approximately 30% (0.27) at four weeks of the treatment. Thus, we further explore the patient distribution according to the achievement of PSA30D at 4 and 12 weeks of the first-line treatment.

**Figure 4:**
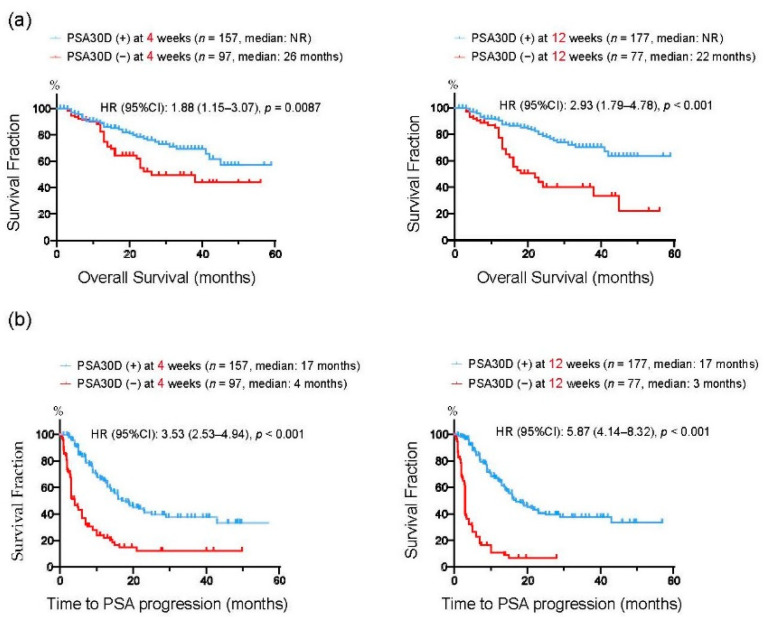
Kaplan-Meier curves for overall survival (OS) (**a**) and time to PSA progression (TTPP) (**b**) from the initiation of the first-line ASIs. In each figure, patients were divided into two groups according to the achievement of >30% PSA decline (PSA30D) at 4 weeks or 12 weeks of the treatment.

We divided 254 mCRPC patients into two groups according to the achievement of PSA30D from the baseline. At four weeks of the treatment, 157 patients (61.8%) had PSA30D from the baseline. Thereafter, 177 patients (69.7%) achieved PSA30D at 12 weeks of the treatment. We next assessed a predictive impact of PSA30D from the baseline at 4 and 12 weeks of the treatment. In agreement with previous studies [22,28,29,30,31,32], the achievement of PSA30D at both 4 and 12 weeks were significantly correlated with an improved OS (4 weeks: *p* = 0.009, 12 weeks: *p* < 0.001: Figure 4a), TTPP (4 weeks: *p* < 0.001, 12 weeks: *p* < 0.001: Figure 4b). To further assess the predictive value of early PSA response, we conducted a multivariate analysis for OS from the initiation of the first-line treatments. A number of variables were found to be independent predictors for OS, including duration of ADT before CRPC (HR: 3.08, 95%CI: 1.67–5.74, *p* < 0.001), ECOG-PS (HR: 3.63, 95%CI: 2.09–6.44, *p* < 0.001), bone metastasis (HR: 4.08, 95%CI: 1.46-14.7, *p* = 0.005), as well as PSA30D at four weeks (HR: 0.48, 95%CI: 0.28–0.84, *p* = 0.009) (Table 2). These data indicated that the achievement of PSA30D as early as four weeks of the first-line ASIs potentially offers a predictive value for OS in mCRPC patients.

To further investigate the clinical value of early PSA response for patients treated with ASIs at the first-line treatment, we stratified patients into four groups according to the achievement of PSA30D at 4 and 12 weeks (Table 3). Of 157 patients (61.8%) who had PSA30D at four weeks of the treatment, most patients (*n* = 147: 93.6%) were consistently offered PSA30D at 12 weeks. On the other hand, of 97 (38.2%) patients without achieving PSA30D at four weeks, 67 (69.1%) patients did not either achieve PSA30D at 12 weeks of the treatment, whereas 30 (30.9%) patients met the criteria for PSA30D at 12 weeks of the treatment.

**Figure 5:**
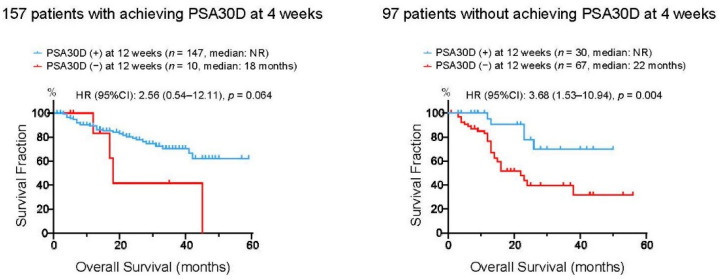
Kaplan-Meier curves for overall survival (OS) from the initiation of the first-line ASIs. Left panel: 157 patients with achieving >30% PSA decline (PSA30D) at four weeks were divided into two groups according to the achievement of PSA30D at 12 weeks of the treatment. Right panel: 97 patients without achieving >30% PSA decline (PSA30D) at four weeks were divided into two groups according to the achievement of PSA30D at 12 weeks of the treatment.

To assess whether these four patient groups stratified by the achievement of PSA30D at 4 and 12 weeks exhibited distinct survival outcomes, we assessed OS in these four groups (Figure 5). Of 157 patients who achieved PSA30D at four weeks of the treatment, patients with achieving PAS30D at 12 weeks appeared to have longer OS than those without achieving PSA30D at 12 weeks (3 years OS rate: 70.5% vs. 41.6% in patients with and without PSA30D at 12 weeks, respectively). Of note, in 97 patients without achieving PSA30D at four weeks, OS in 30 patients (30.9%) who consequently achieved PSA30D at 12 weeks was significantly longer than that in 67 patients (69.1%) without PSA30D in both 4 and 12 weeks (3 years OS rate: 69.7% vs. 39.5% in patients with and without PSA30D at 12 weeks, respectively).

Although the early PSA decline (PSA30D) at four weeks using ASIs as the first-line treatment seemed to be a significant predictor for an improved OS, we noted that 30 of 97 (30.9%) patients, who did not achieve PSA30D at four weeks, but consequently achieved PSA30D at 12 weeks, had a comparable three years OS rate as favorable as 147 patients achieving PSA30D at both 4 and 12 weeks of the treatment (69.7% vs. 70.5% in former and latter, respectively, *p* = 0.642), which prompted us to assess prognostic factors for OS specifically in patients not achieving PSA30D at four weeks of the treatment. To this end, we further performed multivariate analysis for OS in 97 patients who did not achieve PSA30D at four weeks (Table 4). This revealed that duration of ADT < 12 months (HR: 3.45, 95% CI: 1.36–9.25, *p* = 0.008) and ECOG-PS ≥ 1 (HR: 3.98, 95% CI: 1.64–10.32, *p* = 0.002) were identified as independent predictors of shorter OS for mCRPC patients not achieving PSA30D at four weeks of the first-line ASIs.

## 4. Discussion

Precise evaluation of treatment responses for mCRPC is still challenging. Given the number of new agents approved by the FDA in mCRPC setting, identification of early or primary resistance to the treatment is of great value on clinical practice, allowing us to consider an early switch to the next agent and preventing unnecessary treatment that does not benefit for the patient outcome, as well as treatment-related toxicities. However, a recent report that inquired decision-making of clinical management for physicians treating mCRPC had revealed that, despite being considered an important bio-marker, 41.4% of the interviewed physicians disregard PSA changes before 12 weeks of the treatment, whereas the majority of physicians (90.5%) switched treatment based only on clinical progression [33]. This implies that the appropriate timing of next-line treatment is still controversial for mCRPC patients. There have been a number of studies showing that PSA30D after 12 weeks of the treatment is correlated with OS in mCRPC patients [34,35,36]. A recent randomized controlled trial for cabazitaxel (TAXYNERGY study) employed a PSA performance at 12 weeks as a criterion for treatment switch in their trial [37]. Nonetheless, prostate cancer working group 3 (PCWG3) criteria does not encourage treatment decision making based on early PSA changes within the first 12 weeks, since flare reactions of PSA occur in approximately a third of patients treated with taxanes [19,20,21]. On the other hand, the prognostic value of PSA30D at four weeks of the treatment using ASIs has been reported in several studies [22,28,29,30,31,32,38], whereas all those studies included the post-docetaxel population in their analysis. In the present study, we investigated the predictive value of early PSA response (>30% PSA decline: PSA30D) at four weeks of the first-line treatment using AA or Enz for mCRPC patients. The multivariate analysis for predicting OS exhibited PSA30D at four weeks as an independent predictor for a favorable OS.

Despite the strong positive-correlation of PSA changes between 4 and 12 weeks of the first-line ASIs (Figure 2), 30 of 97 patients (30.9%) without PSA30D at four weeks consequently achieved PSA30D at 12 weeks (Table 3). Importantly, those 30 patients had a comparable OS as favorable as 147 patients achieving PSA30D at both 4 and 12 weeks of the first-line ASIs (3 years OS rate of 69.7% vs. 70.5% in 30 and 147 patients, respectively, *p* = 0.642), which implies approximately a third (30 of 97) of patients, who were treated with ASIs as the first-line and did not have an early PSA response (PSA30D) at four weeks, still could benefit from continuing the first-line treatment. A recent report from an international collaborative study analyzing the early PSA response (PSA30D) at four weeks of ASIs included a population of post-docetaxel setting (53.2%) [22]. In their cohort, the proportion consequently achieving PSA30D at 12 weeks in patients without achieving PSA30D at four weeks was 22.0% (100 of 454 patients) compared to 30.9% (30 of 97 patients) in the present study. Unfortunately, survival outcomes in those patients (four groups stratified by the achievement of PSA30D at 4 and 12 weeks) were not exhibited in their report. In the present study, we sought to identify what variables discriminate the patient survival in 97 patients without achieving PSA30D at four weeks of the first-line ASIs by the multivariate analysis (Table 4). The duration of ADT before CRPC < 12 months (HR: 3.45, 95%CI: 1.36–9.25, *p* = 0.008) and ECOG-PS >1 (HR: 3.98, 95%CI: 1.64–10.32, *p* = 0.002) was identified as independent predictors of the shorter OS when no early PSA decline at four weeks was observed in mCRPC patients treated with ASIs as the first-line treatment. These data indicate a possibility of early treatment switch at four weeks of the first-line ASIs when not observing >30% PSA decline (PSA30D) at four weeks, particularly in patients with a modest effect of ADT and poor performance status.

The findings in the present study should be interpreted within several limitations. Firstly, this study was limited by its retrospective design that might have caused selection bias. Secondly, with being devoid of clinical records to define the treatment failure, we could not analyze the treatment outcomes from the second-line treatment, especially in comparing survival outcomes according to sequential therapy [39,40]. Thirdly, the dosage/adherence of the treatment, e.g., relative dose intensity, in individuals was not considered in the present study. Finally, we could not consider biological markers for treatment outcomes, including ARV7 [41].

## 5. Conclusions

This study investigated the predictive value of PSA30D at four weeks of the first-line ASIs for mCRPC patients. PSA changes at four weeks from the baseline were positively correlated with those at 12 weeks. The achievement of PSA30D at four weeks was an independent predictor for OS. Of note, in patients without achieving PSA30D at four weeks (97 of 254 patients: 38.2%), an achievement of PSA30D at 12 weeks was eventually observed in 30.9% of those patients (30 of 97 patients). To identify the variables that discriminate the patient survival in 97 patients without achieving PSA30D at four weeks of the first-line treatment, a multivariate analysis was performed. Duration of ADT before CRPC < 12 months and ECOG-PS >1 were identified as independent predictors for shorter OS for those patients. These data collectively offer a concept of early treatment switch at four weeks of the first-line ASIs when not observing PSA30D at four weeks, particularly in patients with a modest effect of ADT and poor performance status. Large-scale and prospective studies are warranted to prove the result from the present study.

## Figures and Tables

**Table 1:** Clinical Characteristics in 254 mCRPC patients treated with abiraterone: AA (*n* = 119) or enzalutamide: Enz (*n* = 135) at the first-line treatment.

Characteristics	Total (*n* = 254)		1st AA (*n* = 119)		1st Enz (*n* = 135)		*p*-Value
	*n*	%	*n*	%	*n*	%	
Age at CRPC (years)							
mean ± SD	73.0 ± 0.4		72.9 ± 0.7		73.1 ± 0.6		0.880
≤70	94	37.0	40	33.6	54	40.0	0.292
>70	160	63.0	79	66.4	81	60.0	
PSA (ng/ml) at diagnosis							
median (IQR)	179	(40–710)	166	(45–692)	190	(33–715)	0.381
Clinical stage at diagnosis							
T2−T3	131	51.6	55	46.2	76	56.3	0.108
T4	123	48.4	64	53.8	59	43.7	
Gleason sum score at diagnosis							
<9	86	33.9	39	32.8	47	44.8	0.660
≥9	168	66.1	80	67.2	88	55.2	
duration of ADT before CRPC (months)							
≥12	180	70.9	78	65.5	102	75.6	0.079
<12	74	29.1	41	34.5	33	24.4	
ECOG-PS at CRPC							
0	164	64.6	81	68.1	83	61.5	0.273
≥1	90	35.4	38	31.9	52	38.5	
bone mets at CRPC							
−	50	19.7	22	18.5	28	20.7	0.652
+	204	80.3	97	81.5	107	79.3	
lymph node mets at CRPC							
−	135	53.1	64	53.8	71	52.6	0.849
+	119	46.9	55	46.2	64	47.4	
visceral mets at CRPC							
−	215	84.6	101	84.9	114	84.4	0.924
+	39	15.4	18	15.1	21	15.6	
Laboratory data at CRPC							
median (IQR)							
PSA (ng/mL)	10.7	(3.2–31.1)	9.5	(3.1–24.6)	11.1	(3.2–39.0)	0.188
ALP (IU/L)	252	(198–356)	256	(202–381)	247	(161–348)	0.194
LDH (IU/L)	201	(183–239)	199	(182–238)	201	(183–243)	0.350
Hb (g/dL)	12.6	(11.3–13.5)	12.7	(11.4–13.5)	12.6	(9.8–13.4)	0.457
Median OS from the diagnosis of CRPC							
(months)	NR		NR		45		0.325
Median TTPP from the diagnosis of CRPC							
(months)	11		12		11		0.514

mCRPC, metastatic castration-resistant prostate cancer; AA, abiraterone acetate; Enz, enzalutamide; ADT, androgen deprivation therapy; ECOG-PS, eastern cooperative oncology group performance status; mets, metastasis; PSA, prostate-specific antigen; ALP, alkaline phosphatase; LDH, lactate dehydrogenase; Hb, hemoglobin; OS, overall survival; TTPP, time to PSA progression; SD, standard derivation; IQR, interquartile range; NR, not reached.

**Table 2:** Multivariate analysis for OS in 254 mCRPC patients treated with AA or Enz at the first-line treatment.

Variables	Multivariate		
	HR	(95% CI)	*p*-Value
Age at CRPC (years)			
≤70/>70	0.82	(0.47–1.43)	0.484
Clinical stage at diagnosis			
T2−T3/T4	0.81	(0.45–1.45)	0.485
Gleason sum score at diagnosis			
<9/≥9	1.04	(0.67–1.88)	0.876
duration of ADT before CRPC (months)			
≥12/<12	3.08	(1.67–5.74)	<0.001 *
ECOG-PS at CRPC			
0/≥1	3.63	(2.09–6.44)	<0.001 *
bone mets at CRPC			
−/+	4.08	(1.46–14.70)	0.005 *
lymph node mets at CRPC			
−/+	1.56	(0.91–2.67)	0.102
visceral mets at CRPC			
−/+	1.25	(0.62–2.36)	0.500
first-line treatment of CRPC			
AA/Enz	1.55	(0.88–2.77)	0.119
>30% PSA decline (PSA30D) at four weeks			
−/+	0.48	(0.28–0.84)	0.009 *

OS, overall survival; mCRPC, metastatic castration-resistant prostate cancer; AA, abiraterone acetate; Enz, enzalutamide; HR, hazard ratio; CI, confidence interval; ADT, androgen deprivation therapy; ECOG-PS, eastern cooperative oncology group performance status; mets, metastasis; PSA, prostate-specific antigen; * denotes *p* < 0.05.

**Table 3:** Patient distribution according to >30% PSA decline (PAS30D) at 4 and 12 weeks from the initiation of first-line treatment using ASIs (AA or Enz) in 254 mCRPC patients.

Variables		At 12 Weeks	
	PSA30D	(+) (*n* = 177, 69.3%)	(−) (*n* = 77, 30.7%)
at 4 weeks	(+) (*n* = 157, 61.8%)	147 (57.9%)	10 (3.9%)
	(−) (*n* = 97, 38.2%)	30 (11.8%)	67 (26.4%)

PSA, prostate-specific antigen; ASIs, androgen signaling inhibitors; AA, abiraterone acetate, Enz, enzalutamide; mCRPC, metastatic castration-resistant prostate cancer.

**Table 4:** Multivariate analyses for OS in 97 mCRPC patients without >30% PSA decline (PSA30D) at four weeks of the first-line treatment using ASIs.

Variables	Multivariate		
	HR	(95% CI)	*p*-Value
Age at CRPC (years)			
≤70/>70	1.18	(0.49–2.70)	0.694
Clinical stage at diagnosis			
T2−T3/T4	1.02	(0.41–2.45)	0.951
Gleason sum score at diagnosis			
<9/≥9	0.70	(0.27–1.89)	0.480
duration of ADT before CRPC (months)			
≥12/<12	3.45	(1.36–9.25)	0.008 *
ECOG-PS at CRPC			
0/≥1	3.98	(1.64–10.32)	0.002 *
bone mets at CRPC			
−/+	3.66	(0.80–28.81)	0.099
lymph node mets at CRPC			
−/+	1.11	(0.46–2.66)	0.806
visceral mets at CRPC			
−/+	1.14	(0.34–3.13)	0.810
first-line treatment of CRPC			
AA/Enz	1.61	(0.72–3.66)	0.241

OS, overall survival; mCRPC, metastatic castration-resistant prostate cancer; PSA, prostate-specific antigen; ASIs, androgen signaling inhibitors; HR, hazard ratio; CI, confidence interval; ADT, androgen deprivation therapy; ECOG-PS, eastern cooperative oncology group performance status; mets, metastasis; AA, abiraterone acetate; Enz, enzalutamide; * denotes *p* < 0.05.

## Data Availability

No new data were created or analyzed in this study. Data sharing is not applicable to this article.
